# Infant Exploratory Learning: Influence on Leg Joint Coordination

**DOI:** 10.1371/journal.pone.0091500

**Published:** 2014-03-13

**Authors:** Barbara Sargent, Nicolas Schweighofer, Masayoshi Kubo, Linda Fetters

**Affiliations:** 1 Division of Biokinesiology & Physical Therapy, University of Southern California, Los Angeles, California, United States of America; 2 Division of Biokinesiology & Physical Therapy, with Joint Appointments in the Neuroscience and Computer Science Departments, University of Southern California, Los Angeles, California, United States of America; 3 Department of Physical Therapy, Niigata University of Health and Welfare, Niigata, Japan; 4 Division of Biokinesiology & Physical Therapy, Department of Pediatrics, Keck School of Medicine, University of Southern California, Los Angeles, California, United States of America; Scientific Institute Foundation Santa Lucia, Italy

## Abstract

A critical issue in the study of infant development is to identify the processes by which task-specific action emerges from spontaneous movement. Emergent leg action has been studied by providing contingent reinforcement to specific leg movements using an overhead infant-activated mobile, however, there is limited information on the strategies used by infants to support the emergence of task-specific leg action from spontaneous movement. The purpose of this study is to (1) determine the ability of 3 month old infants to learn, through discovery, the contingency between leg action and mobile activation using a virtual threshold, and (2) identify strategies, defined by variance of the end-effectors (feet) and hip-knee joint coordination, used by infants that learned the contingency. Fourteen 3 month old infants participated in 2 sessions of mobile reinforcement on consecutive days. As a group, infants increased the percentage of mobile activation to meet performance criteria on Day 2, but did not meet memory or learning criteria across days. However, five infants learned the contingency based on individual learning criteria. When interacting with the mobile on Day 2 as compared to spontaneous kicking on Day 1, infants who learned the contingency, but not infants who did not learn the contingency, increased variance of the end-effectors (feet) in the vertical, task-specific direction and demonstrated less in-phase hip-knee joint coordination. An important discovery is that infants can discover this very specific contingency, suggesting that this movement behavior (action) can be shaped in future work. This may have implications for the rehabilitation of infants with atypical leg action.

## Introduction

A critical issue in the study of infant development is to identify the processes by which task-specific action emerges from spontaneous movement. Within a Perception-Action framework, infants learn task-specific action through a discovery learning process in which they demonstrate a wide range of exploratory actions to generate information about possible outcomes of actions, then exploit actions which result in outcomes with adaptive value [1], [2]. This exploration-exploitation learning process has been repeatedly described, however, there is limited information on the strategies used by infants to support the emergence of task-specific action from spontaneous movement.

The transition from spontaneous kicking to task-specific leg action serves as a well-studied example. Emergent leg action has been studied by providing contingent reinforcement to specific leg movements using an overhead infant-activated mobile. Infants as young as 2–3 months of age can learn to modify their spontaneous kicking actions when interacting with an infant mobile that reinforces specific leg actions which are within their preferred in-phase movement repertoire, such as increasing the kicking rate of one leg [3]–[5], crossing a specific knee angle [6], [7] or extending the hip and knee together [8].

Our first study using the mobile paradigm supported that 4 month old infants could increase the frequency of kicking and exhibit an out-of-phase coordination pattern of flexing their hip while extending their knee if this movement provided a more direct means to kick a plastic panel that activated an infant mobile [9]. This went beyond previous research in that infants demonstrated that they could move away from their preferred in-phase hip-knee coordination pattern and learn a more out-of-phase hip-knee coordination pattern in response to mobile reinforcement. However, one limitation of our first experimental approach was that the hip-knee joint coordination pattern that was reinforced was constrained by the placement of the panel and was limited in the out-of-phase leg actions that were promoted relative to the extensive movement repertoire that is available to typically developing infants. Another limitation was that we introduced the infants to the causal relationship between contacting the panel and receiving mobile reinforcement by passively guiding the leg of each infant to touch the panel several times.

**Figure 1:**
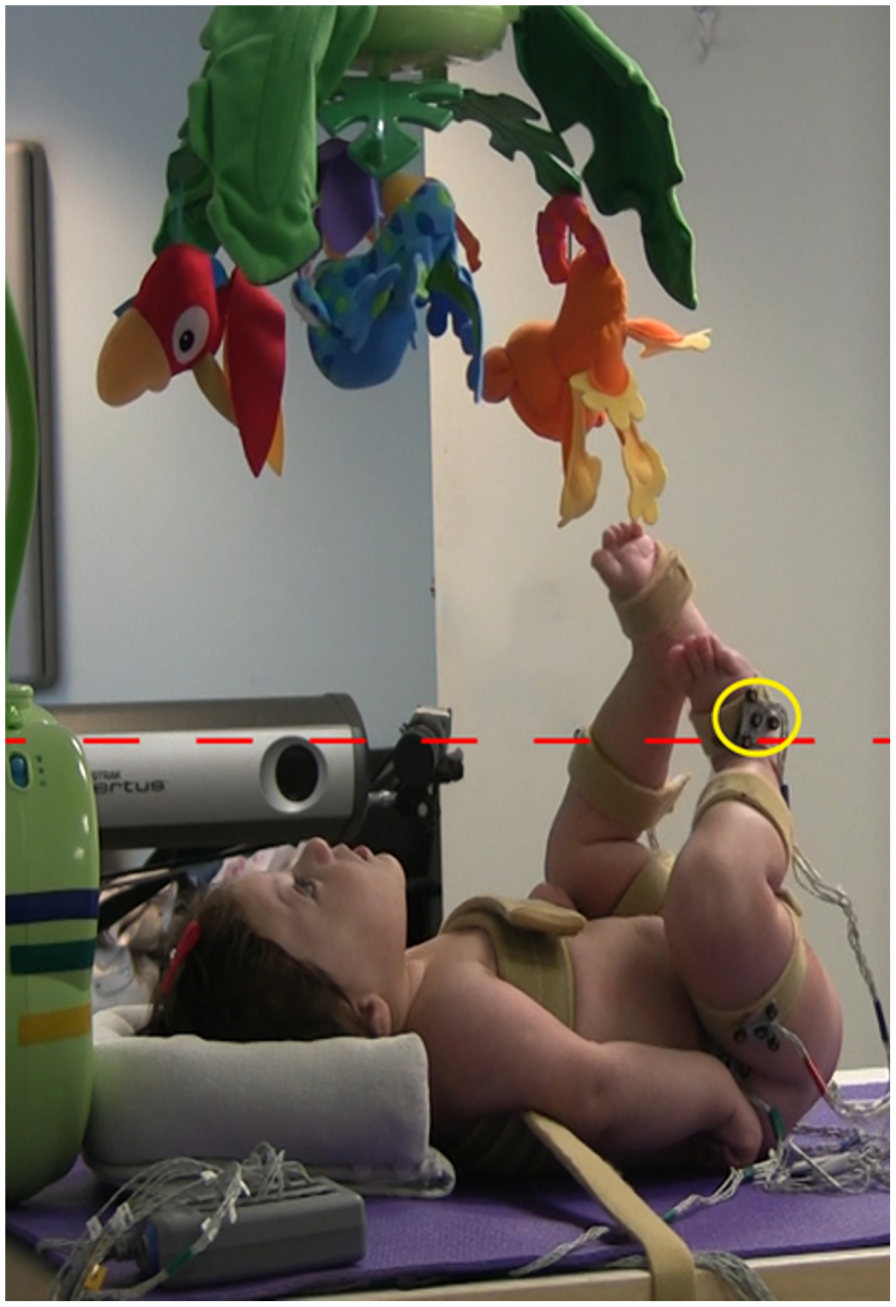
Infant mobile task set-up. Note the infrared light-emitting diode (IRED) placed on each foot (yellow circle) moving vertically to cross the virtual threshold (red dashed line) and activate the mobile. The parent of this child has given written informed consent (as outlined in PLOS consent form) to publish this picture.

In this second study, we have modified our infant-activated mobile paradigm to exploit the discovery learning process. Supine infants activate our mobile by moving their feet vertically across a “virtual threshold” which is individualized to each infant’s baseline spontaneous kicking action (Figure 1, Video S1). What is unique about this modification is that we are ***not*** introducing the infants to the causal relationship between their leg action and mobile reinforcement. Rather, as infants spontaneously move their legs, they ***independently discover*** that their actions activate the mobile. In addition, they discover this contingency without augmented tactile/proprioceptive feedback from a tether connecting their leg to the mobile [3], goniometers attached to their legs [8], or contact with a panel [9]. This better approximates the infant learning process and may contribute to our understanding of the strategies used by infants to perform and learn the task. For example, when exploring the relationship between leg action and mobile activation infants may explore the vertical space by lifting their feet progressively higher, thereby increasing variance of their end effectors (feet) in the vertical, task-specific direction.

Another advantage of our new mobile paradigm, as compared to our previous paradigm, is that it allows a wider range of leg coordination patterns to activate the mobile, yet an individualized placement of the threshold for activation of the mobile promotes an out-of-phase coordination pattern of flexing the hip while extending the knee. Although infants can activate the mobile using an in-phase hip-knee coordination pattern, the use of a less in-phase hip-knee coordination pattern may provide a more efficient means to perform the task.

We have extended our infant mobile paradigm to 2 days in order to evaluate performance, memory, and learning. The traditional mobile paradigm has 3 successive conditions: a non-reinforcement baseline condition (kicking with no mobile reinforcement), a reinforcement acquisition condition (kicking reinforced with mobile activation), and a non-reinforcement extinction condition (kicking with no mobile reinforcement). Performance is assessed each day to determine the extent to which each infant’s response rate (typically, kicking rate) during mobile reinforcement exceeds the baseline rate [10], [11]. Memory is assessed across days to determine the extent to which each infant’s response rate during the baseline condition of the second day exceeds the baseline condition of the first day (this is also called a retention test or baseline ratio) [10]. Learning is assessed across days to determine the extent to which each infant’s response rate during mobile reinforcement on the second day exceeds the baseline rate of the first day (this is also called a relative learning ratio) [12].

In summary, the purpose of this study is to (1) determine the ability of 3 month old infants to learn, through discovery, the contingency between leg action and mobile activation using a virtual threshold, and (2) identify strategies, defined by variance of the end-effectors (feet) and hip-knee joint coordination, used by infants that learned the contingency. We hypothesized that (1) the infants as a group would demonstrate performance of the contingency on the first day and both memory and learning of the contingency on the second day. However, we expected that only a portion of infants would learn the contingency based on the results of previous research [9], [13], [14]. We hypothesized that infants who learned the contingency, as compared to infants who did not learn the contingency, would utilize movement strategies defined by variance of the end effectors (feet) and hip-knee joint coordination. We expected that (2) when leg actions were reinforced with mobile activation, infants who learned the contingency would increase variance in the task-specific (vertical) direction as they explored the vertical space, but would decrease variance once the contingency was learned and they exploited their preferred leg action to activate the mobile. We also expected that (3) when leg actions were reinforced with mobile activation, infants who learned the contingency would exhibit less in-phase hip-knee joint coordination once the task was learned since it would provide a more efficient means to activate the mobile.

## Method

### Participants

Twenty full-term infants participated in data collection at 3 months of age. The infants were born ≥37 weeks gestational age (GA) without birth complications, and were typically developing as per parent report and scores on the motor subtest of the Bayley Scales of Infant and Toddler Development, 3^rd^ Edition [15].

### Ethics Statement

Parents provided written informed consent prior to participation in the study, and families received a small gift for their participation. The Institutional Review Board at the University of Southern California approved the study.

### Procedure

#### Experimental set-up

**Figure 2:**
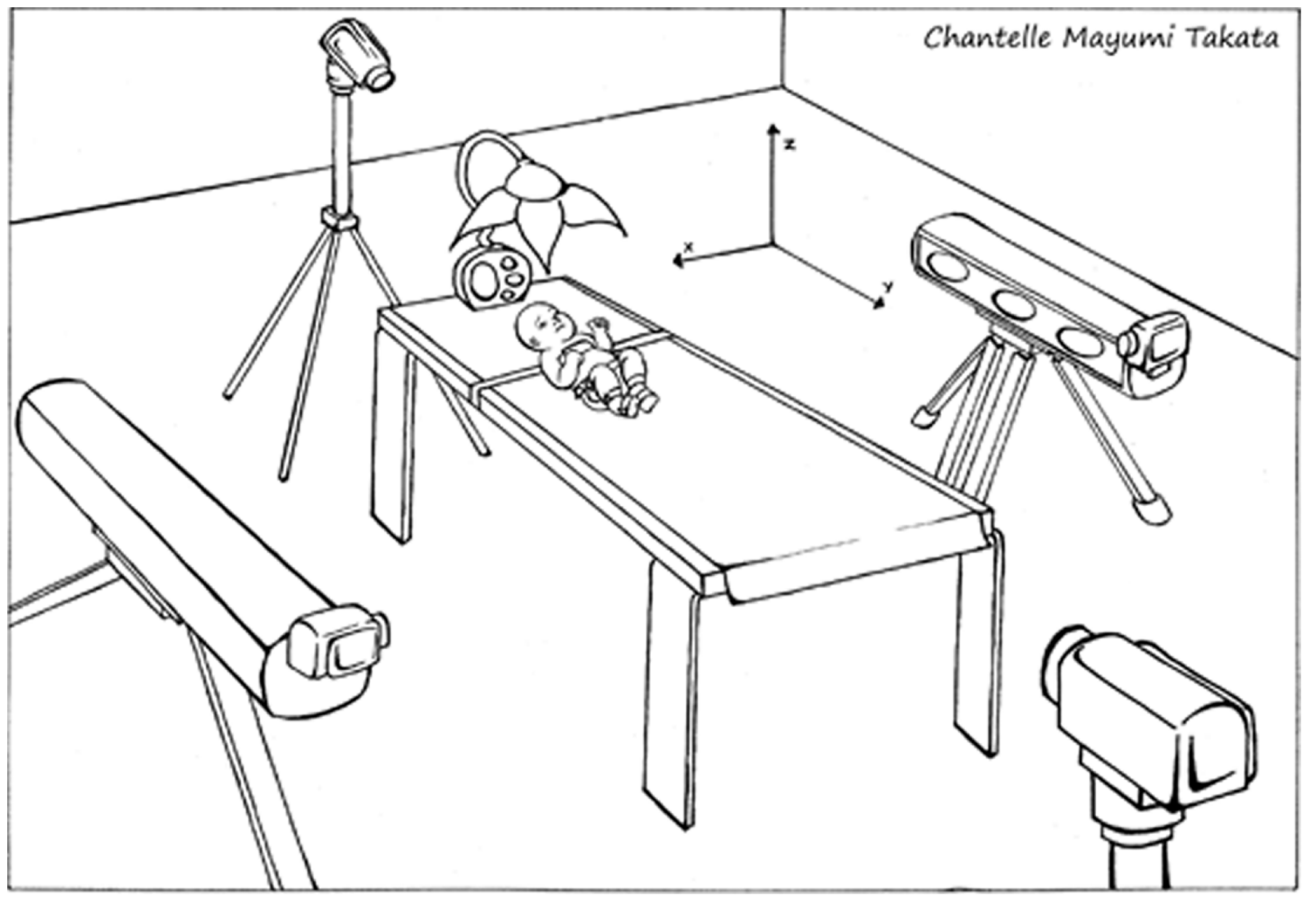
Infant mobile experimental set-up.

Each infant participated in 2 testing sessions conducted on consecutive days at the Development of Infant Motor Performance Laboratory, University of Southern California. During each session, infants were undressed, positioned supine on a table under a conventional infant mobile (Fisher-Price, 2008), and secured to the table using a 4-inch Velcro band placed across the trunk and around the table (Figure 2). The midline position of the head was maintained using a horseshoe-shaped support pillow surrounding the infant’s head. If the infant became fussy or cried during data collection, the parent and investigator provided intermittent visual and verbal contact to reassure the infant. As is consistent with other studies, we decided a priori to exclude infants if they cried for greater than 2 minutes either day of data collection since crying infants are not likely participating in the task [4].

#### Video recording

Infants were video recorded with 3 video cameras that surrounded the testing table (Basler Pylon IEEE1394 cameras using Streampix5 x64 edition multi-camera software) with a right lateral, left lateral, and overhead views of the infant. An additional overhead video camera (Canon HG10) was used to record facial expressions including visual attention.

#### Experimental protocol

**Figure 3:**
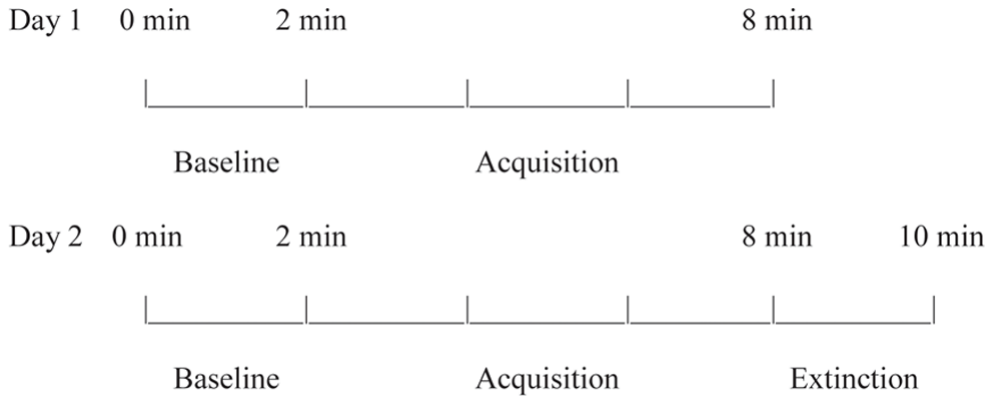
Infant mobile experimental protocol.

On Day 1, a 2-min non-reinforcement condition (baseline) was followed by a 6-min contingent mobile reinforcement condition (acquisition). On Day 2, a 2-min non-reinforcement condition (baseline) was followed by a 6-min contingent mobile reinforcement condition (acquisition) and a 2-min non-reinforcement condition (extinction) Figure 3.

In the baseline condition, the infant was allowed to spontaneously move his legs, but the mobile did not activate in response to the infant’s leg movements. During the acquisition condition, the mobile rotated and music played when the infrared light-emitting diode (IRED) placed on either foot crossed a virtual threshold (Figure 1). The threshold was placed perpendicular to the table and its height was individually determined for each infant as one standard deviation (SD) above the average height of both feet during the Day 1 baseline condition using the following equation:



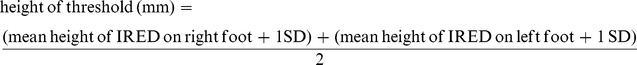



Mobile activation continued for as long as the foot was above the virtual threshold to a maximum of 3 seconds. After 3 seconds, the mobile reactivated only if the infant moved the foot below the virtual threshold, and then moved a foot vertically and again crossed the virtual threshold. This “3 seconds rule” was added because during pilot testing infants would simply hold their feet above the threshold to receive mobile reinforcement. Since we were interested in how infants learned the contingency, specifically whether infants would change variance of the end effector and hip-knee coordination patterns when interacting with the mobile, we added the “3 seconds rule” to encourage leg exploratory movements versus maintenance of a leg posture. The 2516582406-min acquisition condition was divided into three, 2-min intervals (A1, A2, A3) for analyses of performance changes each day. During the extinction condition, the infant was allowed to spontaneously move his legs, but the mobile did not activate in response to the infant’s leg movements.

#### Kinematic data

Throughout the mobile paradigm, three-dimensional lower extremity time-position data were collected at 100 Hz using an Optotrak Certus Motion Capture System (Northern Digital Inc., Waterloo, ON, Canada) with two sensor banks. Each Optotrak sensor bank consisted of three position sensors connected in series to a System Control Unit and a Dell Precision 690 computer.

The two Optotrak sensor banks were placed horizontally approximately 2.5 meters on opposite sides of the testing table (Figure 2). The root-mean-squared (RMS) error in the calibration of the sensor banks was ≤0.3 mm for each data collection session. A global coordinate system was defined in relation to the testing table with the x-axis parallel to the width of the table, y-axis parallel to the length of the table, and z-axis perpendicular to the table.

Rigid marker arrays with 4 embedded IREDs were attached bilaterally to the foot, shank, thigh, and pelvis using Velcro straps. A small plastic piece with 2 embedded IREDs was placed on the sternum using a double-sided sticky EKG collar. After the mobile paradigm, ten individual IREDs were fixed bilaterally to the infant’s skin using double-sided sticky EKG collars at the following locations: lateral midline of the trunk below the tenth rib, greater trochanter of the hip, lateral knee joint line, ankle lateral malleolus, and distal end of the 5^th^ metatarsal. Then, a static calibration trial was collected for each leg by holding the infant’s lower extremity in an extended, anatomical position for 5 seconds. This trial was necessary to define local coordinate systems for each leg segment and define a reference configuration for each body segment in space. All joint angles in this calibration position were defined as zero degrees.

#### Anthropometric data

Each infant was weighed on a digital electric scale (Health-o-meter). The total length of the infant was measured and for both legs the following measures were recorded: circumference at mid-segment of thigh, shank, and foot; width of the knee (at the knee joint line), ankle (at the malleoli), and foot (at the metatarsal heads); and length of the thigh (greater trochanter to knee joint line), shank (knee joint line to lateral malleolus), and foot (medial malleolus to first metatarsophalangeal joint).

### Data Reduction

#### Mobile task data reduction

The dependent measure used to assess performance, memory, and learning of the mobile task is reinforced leg action (RLA). During acquisition, RLA is equal to the duration of mobile activation. During baseline and extinction since the mobile did not activate, we computed the RLA post-hoc using the coordinates of the IRED on each foot that activated the mobile. We computed the total duration of time one or both IREDs were above the virtual threshold subtracting the duration of time in which one or both IREDs were above the virtual threshold for greater than a 3 seconds interval. This replicated the “3 seconds rule” of mobile activation.

Performance, memory, and learning were assessed using group and individual methods as is consistent with previous literature [3], [9], [12], [16]. ***Performance*** of the group was measured each day by determining whether the percent of RLA during any one of the three, 2-min acquisition intervals significantly exceeded the 2-min baseline condition for the same day [3], [6], [9], [16]. For each day, individual infants were identified as having performed the contingency if the percent of RLA during any one 2-min acquisition interval was equal to or greater than 1.5 times the RLA in the 2-min baseline interval [3], [9], [12], [16]. ***Memory*** of the group was defined statistically by determining whether the percent of RLA during the baseline condition Day 2 significantly exceeded the percent of RLA during the baseline condition Day 1 [10], [12], [13]. ***Learning*** of the group was measured statistically by determining whether the percent of RLA during the entire 6-min acquisition condition Day 2 exceeded the percent of RLA during the baseline condition Day 1 [12]. Individual infants were categorized as Learners if the percent of RLA during the entire 6-min acquisition condition of Day 2 was equal to or greater than 1.5 times the baseline condition of Day 1 [12]. Infants that did not meet this individual learning criteria were categorized as Non-Learners.

#### Video data reduction

Video tapes were coded for arousal and attention by an evaluator blinded to group status. The arousal scale is described as: drowsy = 1, alert and inactive = 2, alert and active = 3, fussy = 4, and crying = 5. The attention scale is described as: 0 = not looking at the mobile, 1 = looking at the mobile. This is consistent with previous infant research [7], [17].

#### Kinematic data reduction

Kinematic data were used to determine threshold crossings, as well as compute dependent measures of variance of the end effectors (feet) and hip-knee joint coordination. Position data were converted into 3D coordinates with a direct linear transformation algorithm using Optotrak system software. A custom Matlab (The Mathworks, Inc., Natick, MA) program was used to: (1) interpolate missing position data (maximum of 20 consecutive frames) using a cubic spline, (2) filter position data using a fourth-order Butterworth with a cut-off frequency of 5 Hz as determined from power spectrum analysis, (3) compute joint angles of hip flexion/extension, hip abduction/adduction, hip external/internal rotation, knee flexion/extension, ankle dorsiflexion/plantarflexion, ankle eversion/inversion, and (4) extract kicks. Kick initiation was defined as the onset of a continuous leg movement for which: (a) the infant’s foot moved at least five consecutive frames (1 frame = 10 ms), and (b) the hip and/or knee joint angle change exceeded 11.5° (2 radians) in either flexion or extension [9], [18]–[20]. Kick termination was defined as the frame of peak extension amplitude following a flexion movement or peak flexion amplitude following an extension movement [9], [18].

Joint angles were computed for the hip, knee, and ankle using the method of Söderkvist and Wedin [21] at the following time points: (a) kick initiation, (b) peak velocity of the first segment, (c) joint reversal, (d) peak velocity of the second segment, and (e) kick termination. These points were chosen for analysis because they mark qualitative changes in a movement. Joint angles at these five time points were used either as dependent measures or to compute additional measures.

Variance of the end effector, as defined by the z-variance (vertical, task-specific direction) of the position data of one IRED on the foot rigid marker array, was computed for all kicks extracted for each infant.

Hip-knee joint coordination was defined through the analysis of joint angle correlations and relative phase. Hip and knee flexion and extension joint correlations were computed using Pearson correlation coefficients (*r*) at zero lag between hip and knee joint angle excursions for all kicks extracted for each infant. Hip-knee joint angle correlations were converted to Fisher *Z* scores to allow comparison of correlations (*r*) among infants [9], [18], [20], [22].

Relative phase describes the phase relations between two joint motions. For each kick, joint angle data was time-normalized and continuous relative phase (CRP) was computed for hip and knee flexion and extension from the angular position/velocity data after the method of van Emmerick and Wagenaar [23], [24]. We then analyzed results of the CRP computation at the five time points specified above under “Joint Angles”. Values approaching zero indicate more in-phase coordination; values approaching 180° indicate more out-of-phase coordination. A positive value indicates that the hip is leading the knee in phase space; a negative value indicates that the knee is leading the hip. Since we were interested in the magnitude of out-of-phase coordination, we analyzed the absolute value of the relative phase at each of the five time points.

## Statistical Analysis

### Performance, Memory, Learning of Mobile Task

Performance was assessed each day (Day 1, Day 2) using mixed regression models with a heterogeneous autoregressive (1) covariance structure (ARH1), to test the differences of percent of RLA in any 2-min interval in the baseline, acquisition, and extinction conditions (INTERVAL). To assess memory and learning across days, mixed regression models (ARH1), were used to test the differences of percent of RLA among the 2-min baseline condition, 6-min acquisition condition, and 2-min extinction conditions across days (CONDITION).

### Variance and Coordination Differences of Learners and Non-Learners

Based on the individual learning criteria, infants were separated into those who met criteria (Learners) and did not meet criteria (Non-Learners). To assess variance and coordination changes each day (Day 1, Day 2), mixed regression models with an autoregressive (1) covariance structure (AR1), and group (Learners, Non-Learners) as the between-subject factor were used to test the differences of kick parameters (variance, hip-knee correlation coefficient, relative phase at each of the 5 data points) in any 2-min interval in the baseline, acquisition, and extinction conditions (INTERVAL). To assess memory and learning across days, mixed regression models (AR1) with group (Learners, Non-Learners) as the between-subject factor were used to test the differences of kick parameters (variance, hip-knee correlation coefficient, relative phase at each of the 5 data points) among baseline, acquisition, and extinction conditions across days (CONDITION).

### Arousal and Attention Differences of Learners and Non-Learners

To assess arousal and attention across days, mixed regression models (AR1) with group (Learners, Non-Learners) as the between-subject factor were used to test the differences of arousal and attention among baseline, acquisition, and extinction conditions across days (CONDITION).

For all mixed regression models, the selected covariance structure was chosen because it was consistent with the study design and was the best fit when competing covariance structures were tested using Bayesian Information Criteria and Akaikee’s Information Criteria. All statistical tests were completed using SAS (version 7.0, SAS Institute Inc.) with overall alpha value at 0.05. Preplanned post-hoc comparisons were performed using a Bonferroni correction to adjust for multiple comparisons.

## Results

### Performance, Memory, Learning of Mobile Task

#### Participants

Twenty infants completed data collection at 3 months of age. Two infants were excluded due to equipment difficulties. One infant was excluded due to illness. Three infants were excluded due to excessive crying. Therefore, the study includes 14 infants (7 female, 7 male) between the ages of 99–111 days.

#### Performance

**Figure 4:**
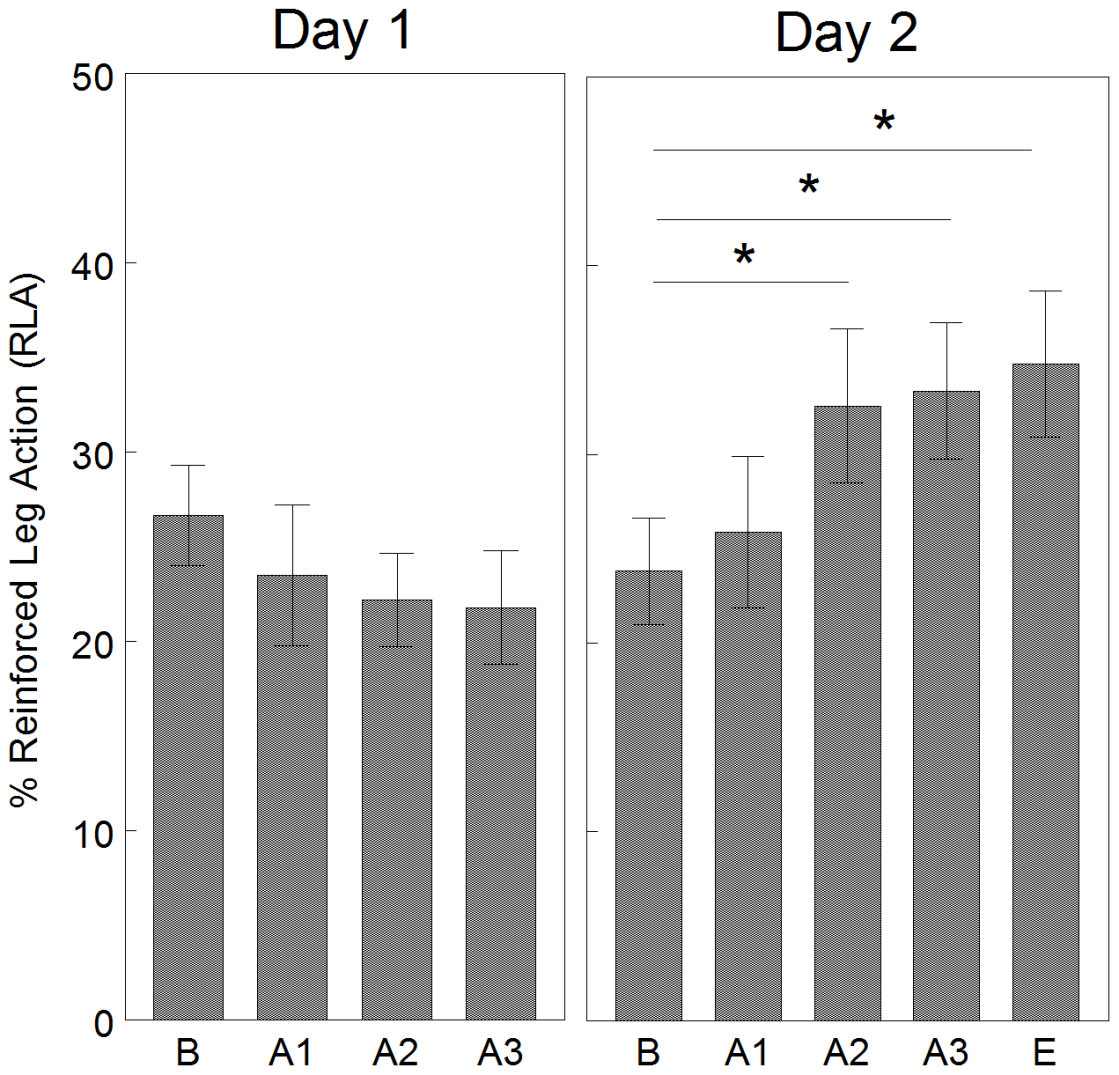
Mean percent reinforced leg action by interval. Infants (n = 14) demonstrated improved performance on Day 2, but not on Day 1. Day 2 there was a significant difference between B and A2, A3, E. * = adjusted p<0.05 Error bars are standard error. A = acquisition, B = baseline, E = extinction.

Percent of RLA during each 2-min interval of Day 1 and Day 2 are graphed in Figure 4. The main effect of interval was not significant for Day 1 [F_(3,39)_ = 0.97, p = 0.42], but was significant for Day 2 [F_(4,52)_ = 2.70, p = 0.041]. Specifically, as compared to baseline of Day 2, the following acquisition intervals were significantly increased: Day 2, acquisition 2 and acquisition 3 (adjusted p<0.05, performance criteria). This can be interpreted as the infants demonstrated improved performance on Day 2, but not on Day 1. When the individual performance of each infant was assessed each day, 4 infants demonstrated performance on Day 1 and 10 infants demonstrated performance on Day 2.

#### Memory and learning

**Figure 5:**
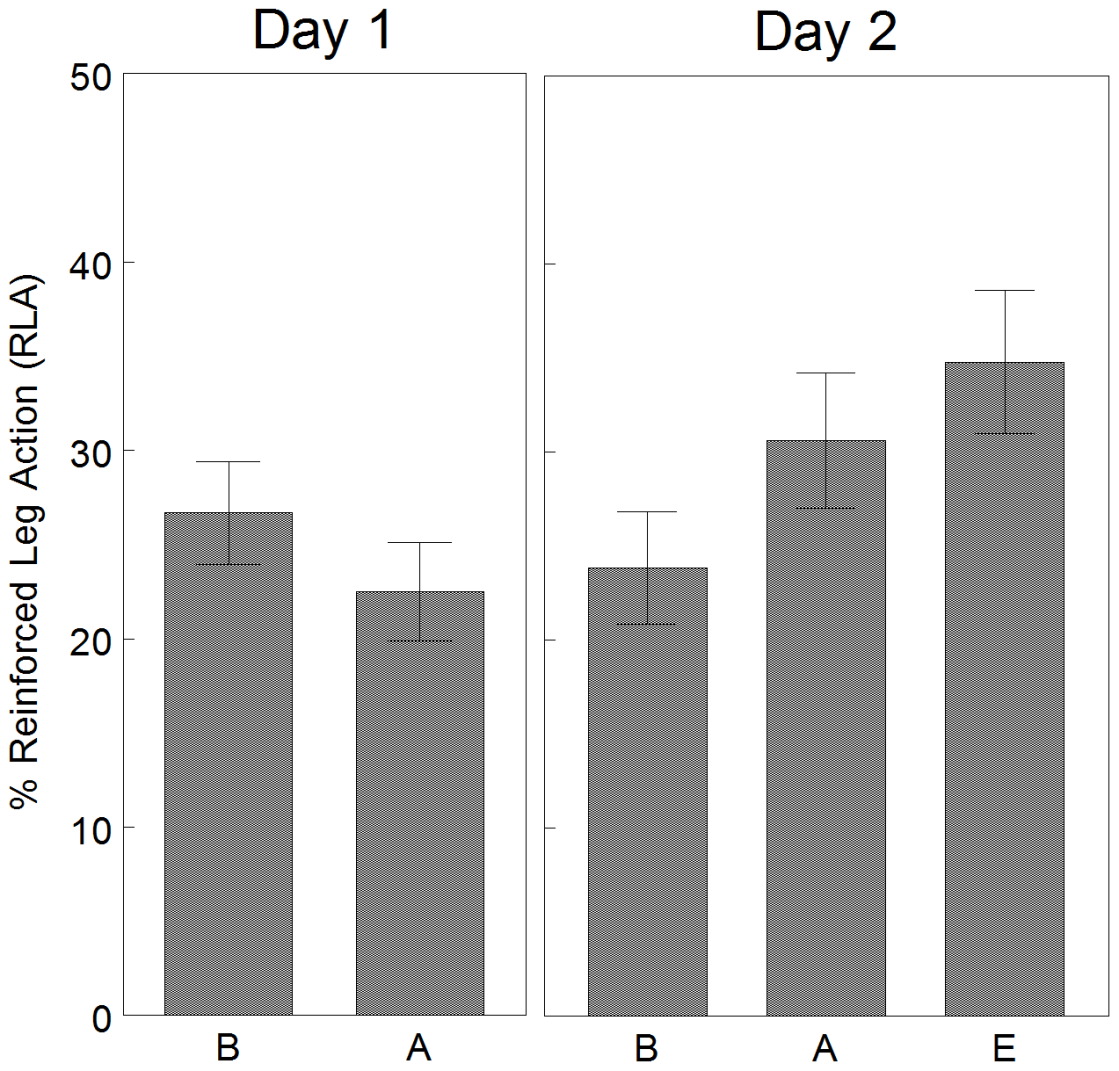
Mean percent reinforced leg action by condition. Infants (n = 14) did not demonstrate a significant change across conditions. Error bars are standard error. B = baseline, A = acquisition, E = extinction.

Percent of RLA across days is graphed in Figure 5. The main effect of condition (2-min baseline, 6-min acquisition, 2-min extinction) was significantly different [F_(4,52)_ = 3.08, p = 0.024]. However, the baseline condition of Day 1 was not significantly different from the baseline condition of Day 2 (adjusted p>0.05, memory criteria) or the acquisition condition of Day 2 (adjusted p>0.05, learning criteria). This can be interpreted as the infants as a group did not remember or learn the contingency across days.

#### Summary of performance and learning results

The infants as a group did not demonstrate increased performance on Day 1 and did not remember or learn the contingency across days, but they did demonstrate increased performance on Day 2.

### Learners and Non-Learners

#### Participants

Since we were interested in determining whether infants who learned the contingency exhibited differences in variance of the end effectors and hip-knee joint coordination when leg actions were reinforced with mobile activation, we separated all 14 infants into infants who learned the contingency (Learners) and infants who did not learn the contingency (Non-Learners). The infants were separated based on the individual learning criteria defined as percent of RLA during the acquisition condition of Day 2 equal to or greater than 1.5 times the baseline condition of Day 1; for example, if the percent of RLA during the 2-min baseline condition of Day 1 was 30%, the percent of RLA of the entire 6-min acquisition condition of Day 2 needed to be equal or greater than 45% for the infant to be classified as a Learner. The Learner group consisted of 5 infants. The Non-Learner group consisted of 9 infants.

#### Kicks

The dependent variables from the kinematic data were computed from the 9,321 leg movements that met the criteria of a kick. Table 1 includes the number of kicks analyzed for each group in each condition.

**Table 1:** Learners versus non-learners: number of kicks analyzed during each condition.

	Day 1		Day 2			Total
	Baseline	Acquisition	Baseline	Acquisition	Extinction	
Learners (n = 5)	279	612	351	1127	513	2882
Non-Learners (n = 9)	656	1855	749	2257	921	6438
Total	935	2467	1100	3384	1434	9320

### Variance Differences of Learners and Non-Learners

#### Performance

**Figure 6:**
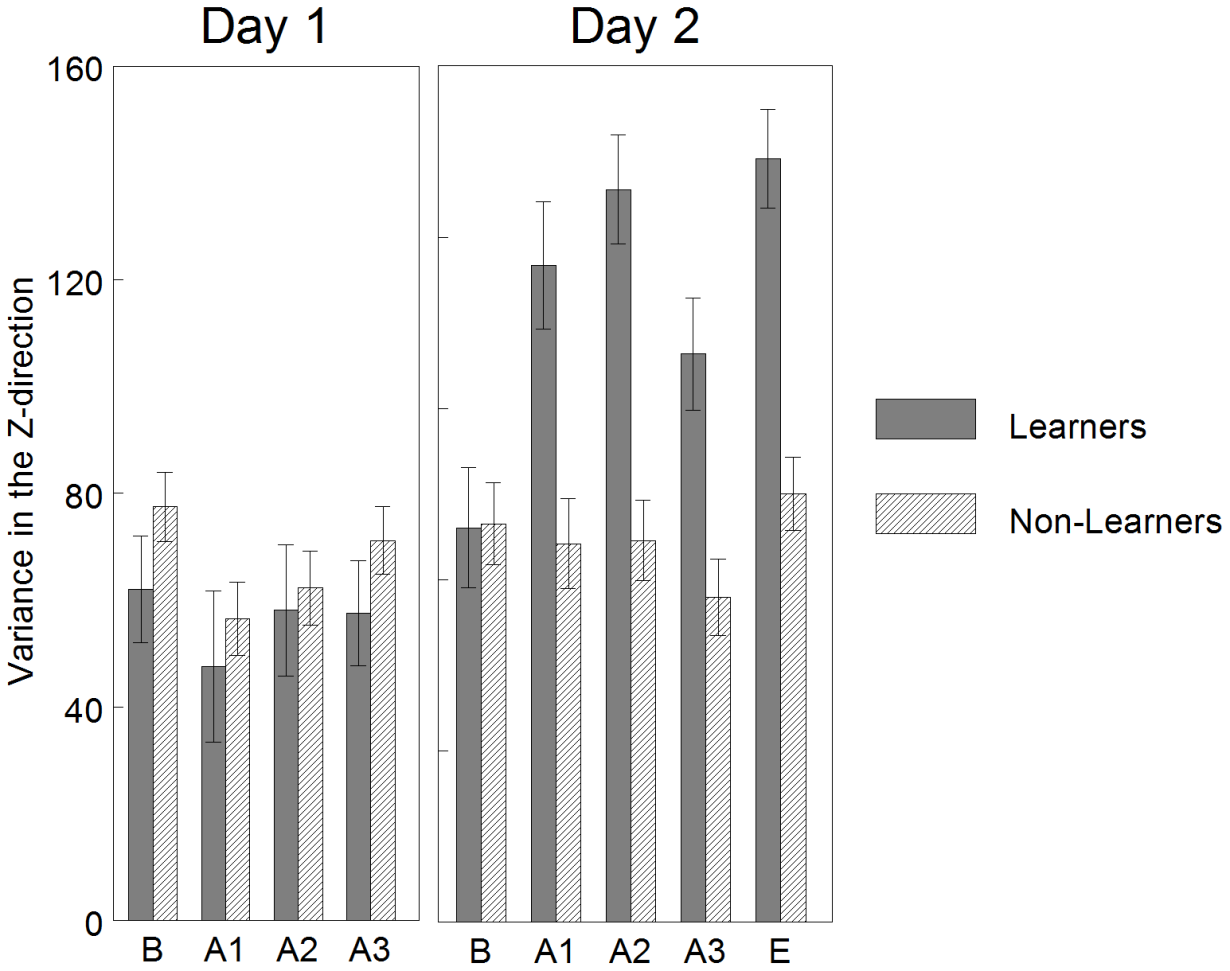
Learners versus non-learners: mean variance in the vertical, task specific direction, by interval. On Day 1, there were no significant differences in variance. On Day 2, between-group variance during each interval, except baseline, was significantly increased in the Learner group as compared to the Non-Learner group. Within-group, the Learner group demonstrated a significant increase in variance in the A1, A2, and E intervals as compared to baseline (performance criteria). Within-group, the Non-Learner group did not demonstrate a significant change between baseline and any acquisition interval (performance criteria). This can be interpreted as the Learner group demonstrated more variance when interacting with the mobile on Day 2, but not Day 1. The Non-Learner group did not demonstrate a change in variance either day. Error bars are standard errors. B = baseline, A = acquisition, E = extinction.

Least squared means and standard error of the variance of the end effector (foot) in the task-specific (vertical) direction across intervals each day is graphed in Figure 6. The interaction of INTERVAL*GROUP was not significant for Day 1 [F_(3,36)_ = 0.15, p = 0.93], but was significant for Day 2 [F_(4,48)_ = 4.14, p = 0.006]. On Day 2, between-group variance during each interval, except baseline, were significantly increased in the Learner group as compared to the Non-Learner group (adjusted p<0.05). Within-group, the Learner group demonstrated a significant increase in variance in the first and second acquisition interval as compared to baseline (adjusted p<0.05, performance criteria). Within-group, the Non-Learner group did not demonstrate a significant change between baseline and any acquisition interval (adjusted p>0.05, performance criteria).

This can be interpreted as the Learner group demonstrated increased variance in the vertical dimension when interacting with the mobile on Day 2, but not Day 1. The Non-Learner group did not demonstrate a change in variance either day.

#### Memory and learning

**Figure 7:**
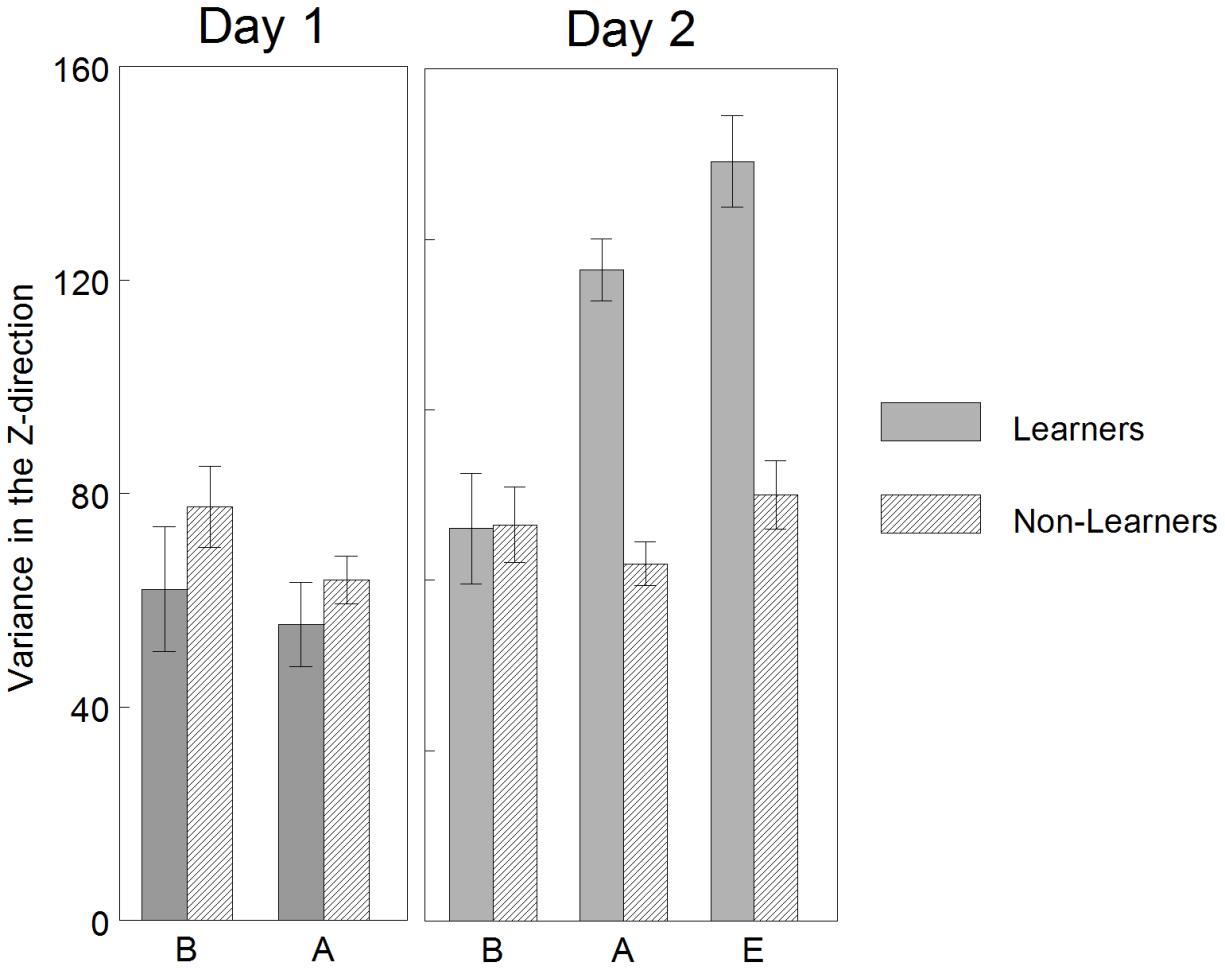
Learners versus non-learners: mean variance in the vertical, task specific direction, by condition. Between-groups, the Learners in comparison to the Non-Learners did not demonstrate a difference in variance during any condition of Day 1, but demonstrated a significant increase in variance during the acquisition and extinction conditions of Day 2. Within-group, the Learners did not demonstrate a significant change in variance between Day 2 baseline and Day 1 baseline (memory criteria), but did demonstrated a significant increase in variance between Day 2 acquisition and Day 1 baseline (learning criteria). Within-group, the Non-Learners did not demonstrate a significant difference in variance between Day 2 baseline and Day 1 baseline (memory criteria) or Day 2 acquisition and Day 1 baseline (learning criteria). This can be interpreted as the Learner group increased their variance when interacting with the mobile on Day 2 as compared to baseline kicking on Day 1. The Non-Learner group did not demonstrate a change in variance across days. Error bars are standard error. B = baseline, A = acquisition, E = extinction.

Least squared means and standard error of the variance of the end effector in the task-specific direction across conditions each day is graphed in Figure 7. Statistical results confirmed an interaction effect of CONDITION*GROUP [F_(4, 48)_ = 13.97, p<0.0001]. Between-groups, the Learners in comparison to the Non-Learners did not demonstrate a difference in variance during any condition of Day 1 (adjusted p>0.05) or the baseline condition of Day 2, but demonstrated a significant increase in variance during the acquisition and extinction conditions of Day 2 (adjusted p<0.001). Within-group, the Learners did not demonstrate a significant difference in variance between Day 2 baseline and Day 1 baseline (adjusted p>0.05, memory criteria), but did demonstrated a significant increase in variance between Day 2 acquisition and Day 1 baseline (adjusted p<0.001, learning criteria). Within-group, the Non-Learners did not demonstrate a significant difference in variance between Day 2 baseline and Day 1 baseline (adjusted p>0.05, memory criteria) or Day 2 acquisition and Day 1 baseline (adjusted p>0.05, learning criteria).

This can be interpreted as the Learner group demonstrated increased variance when interacting with the mobile on Day 2 as compared to baseline kicking on Day 1. The Non-Learner group did not demonstrate a change in variance across days.

### Coordination Differences of Learners and Non-Learners: Correlation Coefficients

#### Performance

**Figure 8:**
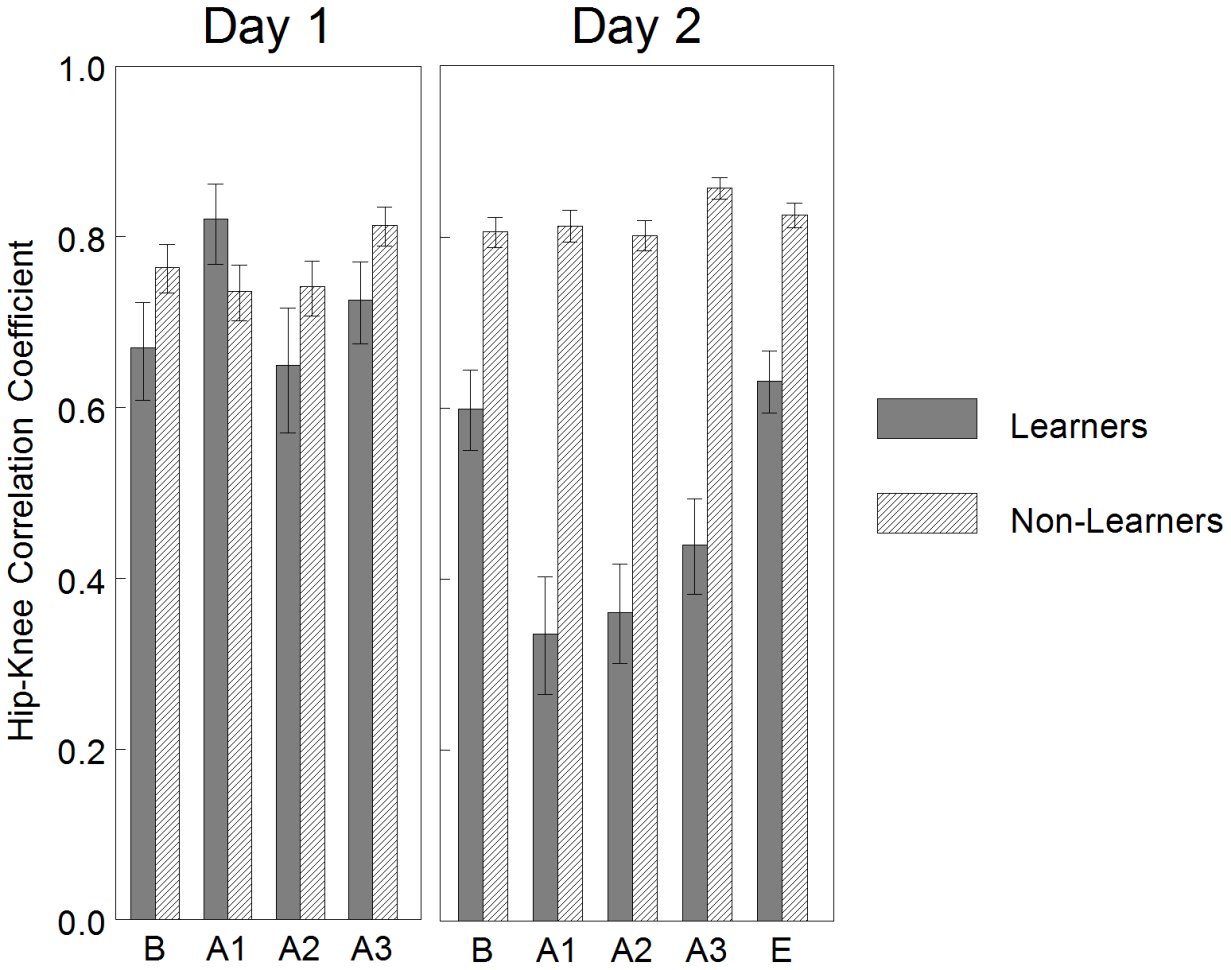
Learners versus non-learners: mean correlation coefficients of hip-knee pair by interval. On Day 1, there were no significant differences in hip-knee correlation coefficients. On Day 2, between-group hip-knee correlation coefficients during each interval were significantly decreased in the Learner group as compared to the Non-Learner group (less in-phase hip-knee joint coordination). Within-group, the Learner group demonstrated a significant decrease in hip-knee correlation coefficients in the first and second acquisition interval as compared to baseline (performance criteria). Within-group, the Non-Learner group did not demonstrate a significant change between baseline and any acquisition interval (performance criteria). This can be interpreted as the Learner group demonstrated less in-phase hip-knee coordination when interacting with the mobile on Day 2, but not Day 1. The Non-Learner group did not demonstrate a change in hip-knee coordination either day. Error bars are standard errors. B = baseline, A = acquisition, E = extinction.

Least squared means and standard error of the hip-knee correlation coefficient across intervals each day is graphed in Figure 8. The interaction of INTERVAL*GROUP was not significant for Day 1 [F_(3,36)_ = 1.89, p = 0.15], but was significant for Day 2 [F_(4,48)_ = 5.27, p = 0.001]. On Day 2, between-group hip-knee correlation coefficients during each interval were significantly decreased in the Learner group as compared to the Non-Learner group (adjusted p = 0.001, less in-phase hip-knee joint coordination). Within-group, the Learner group demonstrated a significant decrease in hip-knee correlation coefficients in the first and second acquisition interval as compared to baseline (adjusted p<0.05, performance criteria). Within-group, the Non-Learner group did not demonstrate a significant change between baseline and any acquisition interval (adjusted p>0.05, performance criteria).

This can be interpreted as the Learner group demonstrated less in-phase hip-knee coordination when interacting with the mobile on Day 2, but not Day 1. The Non-Learner group did not demonstrate a change in hip-knee coordination either day.

#### Memory and learning

**Figure 9:**
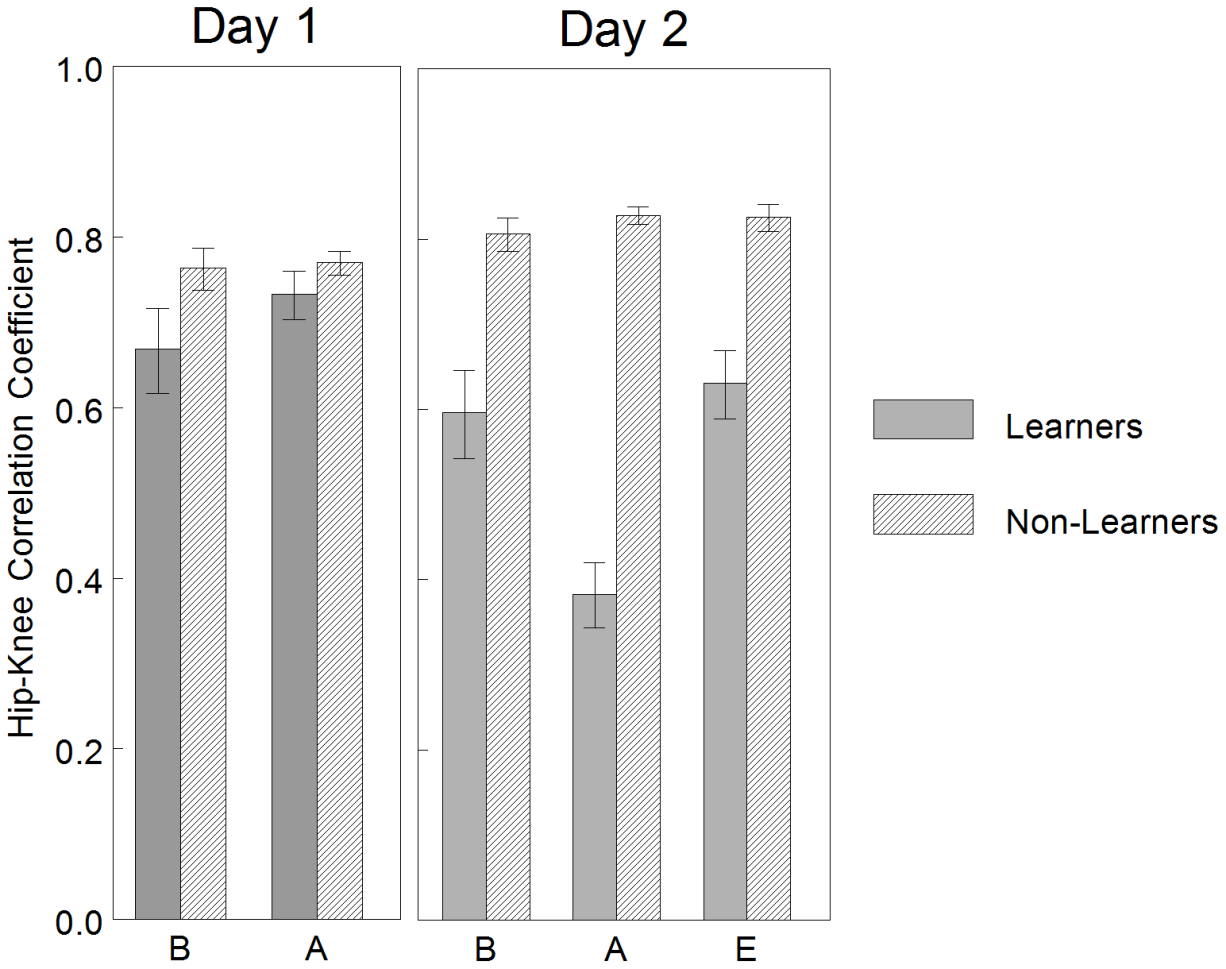
Learners versus non-learners: mean correlation coefficients of hip-knee pair by condition. Between-groups, the Learners in comparison to the Non-Learners did not demonstrate a difference in hip-knee correlation coefficients during any condition of Day 1, but demonstrated a significant decrease in hip-knee correlation coefficients during all conditions of Day 2 (less in-phase hip-knee joint coordination). Within-group, the Learners did not demonstrate a significant decrease in hip-knee correlation coefficients between Day 2 baseline and Day 1 baseline (memory criteria), but did demonstrated a significant decrease in hip-knee correlation coefficients between Day 2 acquisition and Day 1 baseline (learning criteria). Within-group, the Non-Learners did not demonstrate a significant difference in hip-knee correlation coefficients between Day 2 baseline and Day 1 baseline (memory criteria) or Day 2 acquisition and Day 1 baseline (learning criteria). This can be interpreted as the Learner group learned to generate less in-phase hip-knee joint coordination when interacting with the mobile on Day 2 as compared to baseline kicking on Day 1. The Non-Learner group did not demonstrate a change in hip-knee coordination across days. Error bars are standard error. B = baseline, A = acquisition, E = extinction.

Least squared means and standard error of the hip-knee correlation coefficient across conditions each day is graphed in Figure 9. Statistical results confirmed an interaction effect of CONDITION*GROUP for the hip-knee pair [F_(4, 48)_ = 16.97, p<0.0001]. Between-groups, the Learners in comparison to the Non-Learners did not demonstrate a difference in hip-knee correlation coefficients during any condition of Day 1 (p>0.05), but demonstrated a significant decrease in hip-knee correlation coefficients during all conditions of Day 2 (adjusted p<0.001, less in-phase hip-knee joint coordination). Within-group, the Learners did not demonstrate a significant difference in hip-knee correlation coefficients between Day 2 baseline and Day 1 baseline (adjusted p>0.05, memory criteria), but did demonstrated a significant decrease in hip-knee correlation coefficients between Day 2 acquisition and Day 1 baseline (adjusted p<0.01, learning criteria). Within-group, the Non-Learners did not demonstrate a significant difference in hip-knee correlation coefficients between Day 2 baseline and Day 1 baseline (adjusted p>0.05, memory criteria) or Day 2 acquisition and Day 1 baseline (adjusted p>0.05, learning criteria).

This can be interpreted as the Learner group learned to generate less in-phase hip-knee joint coordination when interacting with the mobile on Day 2 as compared to baseline kicking on Day 1. The Non-Learner group did not demonstrate a change in hip-knee coordination across days.

### Coordination Differences of Learners and Non-Learners: Relative Phase

#### Performance

Least squared means and standard error of relative phase at each of the five data points across intervals each day is included in Table 2. On Day 1 between-group, there were no significant differences. On Day 2 between-group, the Learner group, as compared to the Non-Learner group, demonstrated significantly higher values of hip-knee relative phase in all 5 data points during all five 2-min intervals (adjusted p<0.05, less in-phase hip-knee joint coordination). Within-group preplanned comparisons of hip-knee relative phase were generally non-significant when adjusting for multiple comparisons. The only within-group difference was in the Learners; the first acquisition interval of Day 2 relative phase at kick initiation was significantly higher than the Day 2 baseline interval (adjusted p<0.05).

**Table 2:** Learners versus non-learners: relative phase of hip-knee pair by interval.

			Kick Initiation	1^st^ Peak Velocity	Joint Reversal	2^nd^ Peak Velocity	Kick Termination
			mean (SE)	mean (SE)	mean (SE)	mean (SE)	mean (SE)
Day 1	Baseline	Learners	64.6 (4.1)	55.2 (3.9)	54.2 (4.2)	55.8 (4.3)	62.2 (3.9)
		Non-Learners	55.8 (2.7)	44.9 (2.5)	46.5 (2.7)	45.5 (2.8)	54.8 (2.5)
	Acquisition 1	Learners	50.5 (5.8)	46.6 (5.4)	40.6 (5.9)	42.2 (5.9)	53.9 (5.4)
		Non-Learners	58.2 (2.8)	52.7 (2.7)	48.8 (2.9)	48.7 (2.9)	56.6 (2.7)
	Acquisition 2	Learners	60.6 (5.0)	59.9 (4.7)	57.5 (5.1)	59.1 (5.2)	63.2 (4.7)
		Non-Learners	55.7 (2.8)	49.9 (2.7)	47.9 (2.9)	48.2 (2.9)	55.6 (2.7)
	Acquisition 3	Learners	56.8 (4.0)	51.7 (3.8)	51.5 (4.1)	52.2 (4.2)	61.7 (3.8)
		Non-Learners	47.4 (2.6)	42.2 (2.5)	40.7 (2.7)	41.5 (2.7)	52.7 (2.4)
Day 2	Baseline	Learners	64.2 (3.2)*	63.1 (3.0)*	61.8 (3.1)*	64.1 (3.1)*	66.2 (3.0)*
		Non-Learners	46.2 (2.2)	45.7 (2.1)	42.7 (2.1)	40.9 (2.2)	48.6 (2.1)
	Acquisition 1	Learners	84.0 (3.4)* ^,^ †	74.4 (3.2)*	72.2 (3.2)*	72.9 (3.3)*	74.4 (3.2)*
		Non-Learners	47.7 (2.4)	43.3 (2.2)	41.4 (2.3)	38.3 (2.4)	50.3 (2.3)
	Acquisition 2	Learners	74.2 (2.9)*	70.6 (2.7)*	71.6 (2.8)*	71.9 (2.9)*	77.8 (2.7)*
		Non-Learners	47.7 (2.1)	43.9 (2.0)	41.7 (2.1)	40.6 (2.1)	49.8 (2.0)
	Acquisition 3	Learners	75.3 (3.0)*	66.7 (2.8)*	65.9 (2.9)*	65.4 (3.0)*	69.0 (2.8)*
		Non-Learners	45.7 (2.0)	38.5 (1.9)	35.1 (2.0)	33.2 (2.0)	43.1 (1.9)
	Extinction	Learners	60.8 (2.6)*	53.6 (2.5)*	54.5 (2.5)*	54.9 (2.6)*	59.6 (2.5)*
		Non-Learners	44.5 (2.0)	43.5 (1.9)	40.7 (1.9)	39.9 (1.9)	46.5 (1.9)

SE = standard error.

* = Significantly different from Non-Learners, adjusted p<0.05.

† = Significantly different from Learners Baseline Day 2, adjusted p<0.05.

This is consistent with the results from the correlation coefficients that the Learner group, as compared to the Non-Learner group, demonstrated less in-phase hip-knee joint coordination during all intervals of Day 2.

#### Memory and learning

Least squared means and standard error of hip-knee relative phase at each of the five data points across conditions each day is included in Table 3. Between-groups, the Learners as compared to the Non-Learners demonstrated significantly higher values of hip-knee relative phase in all 5 data points during all conditions of Day 2 (adjusted p<0.05, less in-phase hip-knee joint coordination), but not Day 1. Within-group, the Learners did not demonstrate a difference in hip-knee relative phase in any data points between Day 2 baseline and Day 1 baseline (adjusted p>0.05, memory criteria), but did demonstrate significantly higher values in all 5 data points during Day 2 acquisition as compared to Day 1 baseline (adjusted p<0.05, learning criteria). Within-group, the Non-Learners did not demonstrated a significant difference in relative phase in any data points between Day 2 baseline and Day 1 baseline (adjusted p>0.05, memory criteria), and only demonstrated a difference in 2 of 5 data points between Day 2 acquisition and Day 1 baseline (adjusted p<0.05, learning criteria).

**Table 3:** Learners versus non-learners: relative phase of hip-knee pair by condition.

			Kick Initiation	1^st^ Peak Velocity	Joint Reversal	2^nd^ Peak Velocity	Kick Termination
			mean (SE)	mean (SE)	mean (SE)	mean (SE)	mean (SE)
Day 1	Baseline	Learners	64.6 (3.8)	55.4 (3.5)	54.1 (3.7)	55.7 (3.8)	62.1 (3.6)
		Non-Learners	55.8 (2.5)	45.1 (2.3)	46.6 (2.4)	45.6 (2.5)	54.8 (2.3)
	Acquisition	Learners	56.5 (2.5)	52.8 (2.4)	50.9 (2.5)	52.0 (2.6)	60.4 (2.4)
		Non-Learners	53.4 (1.5)	47.9 (1.4)	45.4 (1.4)	45.8 (1.5)	54.8 (1.4)
Day 2	Baseline	Learners	64.2 (3.4)*	63.1 (3.2)*	61.9 (3.3)*	64.2 (3.4)*	66.2 (3.2)*
		Non-Learners	46.2 (2.3)	45.6 (2.2)	42.8 (2.3)	41.1 (2.3)	48.6 (2.2)
	Acquisition	Learners	77.3 (1.9)* ^, †^	70.3 (1.8)* ^,†^	69.8 (1.9)* ^, †^	69.9 (1.9)* ^, †^	73.8 (1.8)* ^, †^
		Non-Learners	47.0 (1.3)^†^	41.7 (1.3)	39.1 (1.3)	37.1 (1.3)^†^	47.4 (1.3)
	Extinction	Learners	60.9 (2.8)*	53.7 (2.6)*	54.5 (2.7)*	55.0 (2.8)*	59.6 (2.6)*
		Non-Learners	44.5 (2.1)	43.6 (2.0)	40.7 (2.0)	39.9 (2.1)	46.5 (2.0)

SE = standard error.

* = Significantly different from Non-Learners, adjusted p<0.05.

† = Significantly different from within-group Baseline Day 1, adjusted p<0.05.

This can be interpreted as the Learner group, but not the Non-Learner group, demonstrated less in-phase hip-knee joint coordination when interacting with the mobile on Day 2 as compared to the baseline condition of Day 1. These results are consistent with results from the correlation coefficients.

### Relation between Learning and Coordination Changes

**Figure 10:**
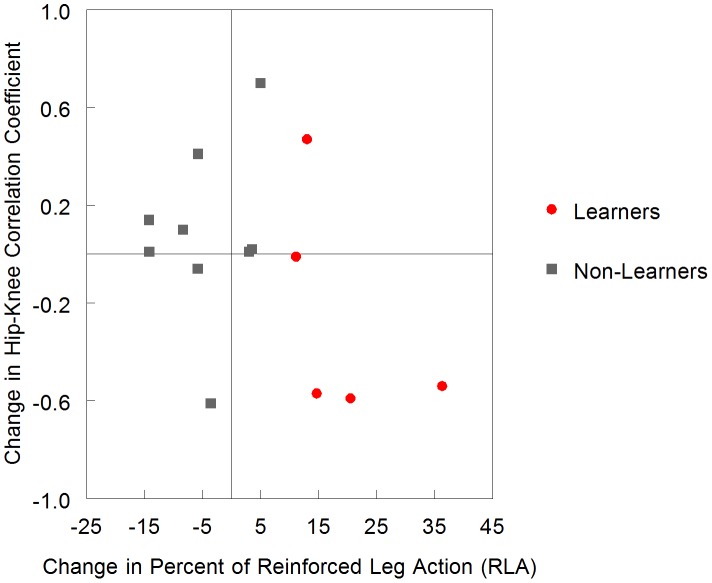
Scatterplot of change in percent of reinforced leg action by change in hip-knee correlation coefficient. Difference in percent of reinforced leg action (RLA) between Day 2 acquisition condition and Day 1 baseline (learning criteria) by difference in hip-knee correlation coefficient between Day 2 acquisition condition and Day 1 baseline.

To depict the relation of learning and coordination changes, we plotted the change in percent of RLA from the Day 2 acquisition condition to the Day 1 baseline condition (our individual learning criteria) and difference in hip-joint coordination from the Day 2 acquisition condition to the Day 1 baseline condition in Figure 10. Three of 5 infants in the Learner group decreased their hip-knee correlation coefficient when interacting with the mobile on Day 2 as compared to baseline spontaneous kicking, whereas only 1 of 9 infants in the Non-Learner group decreased his hip-knee correlation coefficient.

### Arousal and Attention Differences between Learners and Non-Learners

#### Arousal

The interaction of CONDITION*GROUP [F_(4,48)_ = 0.09, p = 0.98] and the main effect of GROUP [F_(1,12)_ = 0.10, p = 0.76] were not significant. The main effect of CONDITION was significant [F_(4,48)_ = 7.62, p<0.0001]. Infants in both groups were classified as alert (a score of 2 or 3 on the arousal scale) for 92–98 percent (SE<5 percent) of the experimental time except during the extinction condition of Day 2 which was 71 percent (SE 5 percent), significantly different from all other conditions (adjusted p<0.05). This can be interpreted as there was no difference between the Learner and Non-Learner groups in terms of their arousal during the mobile paradigm, however, both groups of infants were significantly more fussy or crying during the extinction condition as compared to baseline and acquisition conditions.

#### Attention

The interaction of CONDITION*GROUP [F_(4,48)_ = 3.65, p = 0.01] was significant. In both groups, the mean percent of time during each condition in which the infant was looking at the mobile ranged from 94–99 percent (SE 3 percent), except during extinction when the mean percent of time was 86 percent in the Learner group. When adjusting for pre-planned multiple comparisons, there were no between or within-group differences. This can be interpreted as there was no difference between the Learner and Non-Learner groups in terms of their attention during the mobile paradigm.

## Discussion

We hypothesized that the infants would demonstrate performance of the contingency on the first day and both memory and learning of the contingency on the second day. Contrary to our hypothesis, the infants as a group demonstrated improved performance on the second day, not the first day as reported in previous research. In addition, they did not demonstrate either memory or learning of the contingency across the two days. There are two possible explanations for these results. First, in our mobile paradigm, the infants ***independently*** discover the contingency as their exploratory leg actions activate the mobile. In previous paradigms, infants were often shown that the mobile moved either through the investigator passively guiding the leg [9], a shaping reinforcement schedule [8], or activation of the mobile by the investigator at the beginning of the acquisition condition [6]. Our research is grounded within a Perception-Action framework, thus we allowed infants to ***independently*** discover the contingency without external assistance. We believe our experiment more closely approximates the infant learning environment although it may have resulted in infants requiring more time to demonstrate performance and learning of the contingency. When we assessed the individual performance of each infant on each day, more than twice as many infants improve performance on Day 2 compared to Day 1 (10 infants on Day 2 and 4 infants on Day 1). Therefore, we believe that if our study had been extended an extra day, a much higher proportion of infants would have demonstrated learning across days.

This raises the question for infant learning as to the benefit of demonstration versus self-discovery. If the immediate task is to be learned, then demonstration of a contingency or aspects of the contingency (e.g. that the mobile can move) may be the most rapid method for task learning. It is not clear that this type of learning would generalize to other tasks. However, discovery learning, such as provided in our mobile paradigm, may provide an opportunity for learning a process that could be generalized to the learning of other tasks. This could be empirically addressed with an experimental protocol that first offers a contingency task to be learned. One group of infants could be shown or guided through the contingency while a second group was left to independently discover the contingency. After the initial contingency is introduced and learned additional contingency tasks are then tested. We hypothesize that the time needed to learn subsequent contingency tasks would be reduced for the group “learning to learn” through discovery actions in comparison to the group who is guided through the task. Adolph and colleagues have conducted a series of studies in which infants were exposed to variable and novel challenges of balance and locomotion, such as descending slopes, spanning gaps, and crossing bridges [25]. Overall, they found that when infants first acquired a new posture, such as walking, they did not take into account the limits of their abilities relative to risky environmental features, for example they would attempt to walk down steep slopes [26]. Over weeks of walking experience, their responses became more adaptive and they would attempt safe slopes and refuse steep slopes. In fact, infants with walking experience could even respond adaptively to the novel experience of descending a slope wearing either a lead-weighted or feather-weighted shoulder pack. Experienced walkers demonstrated that they generalized their knowledge of slopes by immediately walking down relatively steep slopes with the feather-weighted shoulder packs, but refusing to walk down the same slope with the heavier, lead-weighted shoulder packs [27]. This demonstrates that they were able to generalize their knowledge of walking down slopes to a novel task utilizing shoulder packs of varying weight. They had “learned to learn.”

Another possible explanation why infants did not perform our task the first day is that our paradigm requires more specified control of leg action than is typically required in the mobile paradigm. In previous paradigms, in order to demonstrate performance of the contingency, infants simply needed to increase the frequency of leg actions that were within their preferred movement repertoire of in-phase intralimb coordination, such as increasing kicking rate [3]–[5], demonstrating flexion or extension of the knee [6], [7] or demonstrating in-phase flexion or extension of the hip and knee joints [8]. In our mobile paradigm, a less in-phase hip-knee joint coordination pattern provided a more efficient means to activate the mobile. This is supported by the fact that 60 percent of infants who demonstrated learning, as compared to 11 percent of infants who did not demonstrate learning, demonstrated less in-phase hip-knee coordination when interacting with the mobile on the second day. The change in coordination pattern may have been difficult for the infants to independently discover in two, 6-minute testing sessions. To support an infant’s ability to independently discover the contingency, the threshold to activate the mobile could be modified as the experiment unfolds. Infants who were “non-learners” in our current paradigm may be able to learn if the threshold is lowered such that more of their kicks activate the mobile. Once this contingency is established, the threshold could be raised such that the coordination pattern then shifts to a less in-phase pattern. This may provide the “just right challenge” necessary to learn the task.

### Strategies Used by Learners

Our second hypothesis was that infants who learned the contingency, as compared to infants who did not learn the contingency, would increase variance in the task-specific (vertical) direction as they explored the relationship between their leg action and mobile activation, but would decrease variance once the contingency was learned and they exploited their leg action to activate the mobile. We found that infants who learned the contingency increased variance of their feet (end effector) in the task-specific direction during the first and second acquisition intervals of Day 2. These results support that infants use their end effectors to explore the task-specific space as they discover whether their actions result in interesting sensory experiences from the environment. We did not find significant evidence of a decrease in task-specific variance once the contingency was learned, however, the variance during the third acquisition interval of Day 2 was beginning to decrease as compared to the first and second acquisition intervals. A longer paradigm may be required to investigate the variance change of the end effector with exploration and exploitation.

Our third hypothesis was that infants who learned the contingency, as compared to infants who did not learn the contingency, would exhibit less in-phase hip-knee joint coordination when leg actions were reinforced with mobile activation. This hypothesis was confirmed using both an analysis of correlation coefficients and relative phase. These results support that infants can change their coordination patterns if they provide a more efficient means to elicit an interesting sensory experience, in this case, activation of the mobile. This change in movement pattern through exploratory learning should be considered when supporting the learning of infants. Using careful consideration of the infant/environment interface, learning environments can be constructed such that desired movement patterns are discovered by the infant as constructed environments are explored.

### Contribution to the Literature

This study extends previous work examining learning of young infants using the mobile paradigm. Unique to this study is that infants self-discovered the contingency between mobile activation and their leg movement without demonstration of aspects of the contingency [6], [8], [9] or augmented sensory input [3], [8], [9]. In addition, the study provides insight into how new coordination patterns emerge during task-specific action. Infants who learned the contingency appeared to use their end effectors (feet) to explore the vertical, task-specific space to elicit mobile reinforcement. This resulted in the use of a less in-phase hip-knee joint coordination pattern, which is not within the preferred movement repertoire of young infants. It is hypothesized that continued practice interacting with the mobile may increase the strength and movement control of the lower extremities resulting in the less in-phase hip-knee joint coordination pattern becoming part of an infant’s preferred movement repertoire.

### Clinical Relevance

Further research is necessary to determine whether infants at high risk for movement disorders can change their coordination patterns when participating in discovery learning paradigms. Children with spastic cerebral palsy (CP) demonstrate excessive in-phase hip-knee joint coordination, which affects their functional mobility [28]–[30]. Preterm infants are at high risk for white matter damage, which has been associated with the development of spastic CP [31], [32]. Some preterm infants with white matter damage also demonstrate excessive in-phase hip-knee joint coordination [22], [33]. The next step in this research line would be to determine whether preterm infants with white matter damage, at increased risk for CP, can generate less in-phase hip-knee joint coordination when participating in discovery learning paradigms.

### Limitations

A limitation of the present study is the small sample size of infants, particularly of infants who learned the contingency across days. Nine infants were classified as Non-Learners and 5 as Learners based on our individual learning criteria. We found statistically significant differences in variance of the end-effectors (feet) and hip-knee joint coordination between the leg movements of the Learner and Non-Learner groups. Although these findings were based on a small number of infants, they were computed from an analysis of thousands of kicks from each group of infants (2,882 kicks from Learners and 6,438 from Non-Learners). In addition, these differences in variance and hip-knee joint coordination were not observed on the first day of the experiment, but only during the second day of the experiment when the Learners demonstrated that they had learned the contingency based on the percent of RLA, essentially the amount of time the mobile was activated.

## Conclusion

The results of this study highlight that some infants can self-discover the contingency between mobile activation and leg movement in only 2 days. Those that learned the contingency increased variance of the end-effectors (feet) in the vertical, task-specific direction and demonstrated less in-phase hip-knee joint coordination when interacting with the mobile. An important discovery is that infants can self-discover this very specific contingency, suggesting that this movement behavior (action) can be shaped in future work.

## Supporting Information

Video S1 **Infant mobile task.** Note whenever the infant moves her foot vertically to cross the virtual threshold, the mobile activates. The parent of this child has given written informed consent (as outlined in PLOS consent form) to publish this video.
(MP4)
